# Patient outcomes of monotherapy with hypofractionated three-dimensional conformal radiation therapy for stage T2 or T3 non-small cell lung cancer: a retrospective study

**DOI:** 10.1186/s13014-016-0582-1

**Published:** 2016-01-19

**Authors:** Masakuni Sakaguchi, Toshiya Maebayashi, Takuya Aizawa, Naoya Ishibashi, Shoko Fukushima, Osamu Abe, Tsutomu Saito

**Affiliations:** Department of Radiology, Nihon University School of Medicine, 30-1, Oyaguchi Kami-cho, Itabashi-ku, Tokyo 173-8610 Japan; Sonodakai Radiation Oncology Clinic, 4-1-12, Takenotsuka, Adachi-ku, Tokyo 121-0813 Japan

**Keywords:** NSCLC, Radiation therapy, 3D-CRT, Radiotherapy

## Abstract

**Background:**

Hypofractionated three-dimensional conformal radiation therapy (3D-CRT) is a treatment option for patients with early-stage non-small cell lung cancer (NSCLC) who are medically unable to tolerate surgery and who are not amenable to treatment with stereotactic body radiotherapy. This study assessed the efficacy and safety of 3D-CRT as a monotherapy in patients with localized stage T2 or T3 NSCLC.

**Methods:**

This retrospective study consisted of 29 patients (20 males) aged 56–89 years (median, 76 years) with histologically confirmed NSCLC who underwent 3D-CRT between 2005 and 2014.

**Results:**

The median duration of patient observation was 17.0 months (range, 1.0–64.0 months). Complete and partial responses occurred in 13.8 and 44.8 % of patients, respectively, and the overall response rate was 58.2 %. Meanwhile, the 1- and 3-year survival rates were 65.8 and 33.8 %, respectively. In T2 NSCLC, the median survival time (MST) was 12 months, and the 1- and 3-year survival rates were 62.4 and 21.4 %, respectively. In T3 NSCLC, the MST was 17 months, and the 1- and 3-year survival rates were 72.9 and 48.6 %, respectively. Severe toxicities (Common Terminology Criteria Grade 3) were not observed. The mean biologically effective dose required to improve local control exceeded 80 Gy (range, 67.2–96.0 Gy).

**Conclusion:**

These findings support a role for 3D-CRT as a treatment option for patients who refuse or could not tolerate surgical therapy with early-stage NSCLC. Although this was a small, retrospective study, it may form the basis for future, larger controlled studies on 3D-CRT as a monotherapy for NSCLC.

## Introduction

Three-dimensional conformal radiation therapy (3D-CRT) delivers radiation to tumors while sparing surrounding normal tissue structures. The use of patient-specific 3D images allows for treatment planning in a manner that is distinct from conventional radiotherapy techniques. The complementary use of computed tomography (CT) and magnetic resonance imaging (MRI) of the lungs is performed to define the area of lung tumors. Identifying areas of tumor and normal lung tissue allows for selective and concentrated radiation therapy with less damage to normal lung tissue. Surgical resection is one treatment method for localized non-small cell lung cancer (NSCLC), and good outcomes are achieved with this method in stage I or II NSCLC [1–3]. While surgery is recommended for some patients with localized NSCLC, the proportion of operable patients declines with age [4, 5]. Radiation therapy is selected if pulmonary function is poor, if surgery is contraindicated due to the patient’s general condition, or if the patient refuses surgery. However, satisfactory results for patients with localized NSCLC have not been obtained with conventional irradiation at a dose of 2 Gy given in a single fraction [6–9]. In conventional radiation therapy, therapeutic effects are obtained on the basis of differences in the degrees of damage and recovery between normal and cancer cells. Damage to normal cells can be reduced by employing this irradiation method. Also, the treatment duration is prolonged to increase the dose, which may increase the risk of tumor regrowth. Further drawbacks include a low single radiation dose that reduces the anti-tumor effects and changes in the characteristics of cancer cells, especially their sensitivity to radiation, when the treatment duration is prolonged. For this reason, the duration of radiation treatment should be kept short, and the radiation dose should be increased for improved tumor control. Stereotactic body radiotherapy (SBRT) has also been established as a treatment option for localized NSCLC [10, 11]. SBRT results in improved patient outcomes compared with conventional irradiation, with results that rival surgery [12–14]. However, SBRT requires greater precision and accuracy than conventional radiotherapy, and it should be conducted according to a strict protocol [15]. Furthermore, patient characteristics such as tumor size, site, and general physical condition can make performing SBRT difficult. In such instances, hypofractionated 3D-CRT is an alternative treatment option [16, 17]. Some studies of hypofractionated radiotherapy (HFRT) using various radiation dose schedules have reported improved outcomes beyond those achieved with conventionally fractionated radiotherapy [16–19]. High-dose irradiation with a biologically effective dose (BED) of 100 Gy or more enables good localized disease control in SBRT, and thus, it may be assumed that not necessarily a mere increase in the total radiation dose, but increasing the BED as well will also lead to good localized control in 3D-CRT [12]. Previous reports indicated that when the radiation dose is increased to 4 Gy delivered in a single fraction, there are no severe adverse events and good localized control can be achieved [20]. There are few reports that describe 3D-CRT to treat patients with localized NSCLC without the use of combined anticancer agents. At our institution, we have recently administered radiation treatment to patients with stage T2 or T3 NSCLC, in whom we increased the radiation dose to more than 5 Gy delivered in a single fraction. In these patients, the radiation field is defined as the tumor plus margin, and the use of 3D-CRT is limited to patients for whom elective nodal irradiation was not conducted. This study investigated the safety and efficacy of 3D-CRT as a monotherapy in patients with T2 or T3 localized NSCLC and examined the factors influencing patient prognosis.

## Methods

### Patients studied

This retrospective study included 29 patients (20 males) aged 56–89 years (median, 76 years) with histologically confirmed NSCLC who received hypofractionated 3D-CRT between January 2005 and June 2014. Patients with severe heart and lung disease who required regular oxygen treatment were excluded. SBRT was indicated for patients with peripheral lung malignancies measuring 3 cm or less with a forced expiratory volume (FEV)_1.0_ of 800 ml or more and a performance status (PS) of 0 to 1 and also for patients with lung cancer located 2 cm or more from the great vessels in the hilar region. Although there was no strict age limit, a cut-off of 85 years was used as the upper age limit for patients with PS 2. Even in patients who met the indications for SBRT, 3D-CRT was selected when they could not adequately synchronize respiration because lesions were located in the lower pulmonary lobes and there was marked fluctuation.

When postoperative FEV_1.0_ was expected to be 800 ml or more, surgery was indicated in patients with PS 0 to 1 and localized lung cancer without complications. Although combination treatment with anticancer agents and radiation was the standard of care even for patients lacking indications for SBRT or surgery, the most common reason for selecting radiotherapy alone was renal impairment, followed by patient refusal and poor PS.

### Staging investigations

Staging was performed using contrast-enhanced CT and positron emission tomography (PET), according to the recommendations of the current staging guidelines for NSCLC [21]. Tumors located no more than 2 cm from the pulmonary hilum were classified as the pulmonary hilum type. In seven patients, staging was performed using contrast-enhanced CT without PET. In addition to standard blood tests, the presence of NSCLC tumor markers was assessed, including monoclonal antibodies to squamous cell carcinoma antigen, carcinoembryonic antigen, and CYFRA 21.1 (a variant of cytokeratin 19) [22]. Radiography of the chest, MRI of the brain, and bone scintigraphy were performed. Staging, therapeutic effect, and the presence or absence of recurrence were determined by a radiologist, a respiratory medicine specialist, and a radiotherapist.

### Radiation therapy

The treatment plan was performed with CT using a long scan time (3 s). Tumor motion was accounted once at the time of CT simulation. Scans were assessed in 3-mm sections at the lesion site and 10-mm sections elsewhere. The gross tumor volume was the volume of the area occupied by the tumor as measured by image diagnosis. However, because a long scan time was used, the clinical target volume (CTV) was used to define the visible range of CT. The internal target volume (ITV) was the CTV plus the tumor margin for any organ movement. The ITV included a 5–7 mm ‘set up’ margin to establish the planning target volume (PTV). The radiation field was defined as the PTV plus a 5-mm leaf margin. Using a 6-MV X-ray beam, multifield irradiation to more than four fields (all noncoplanar irradiation) was administered under resting respiration. Each beam was created using PTV along the path of the beam with a margin. Additional techniques (for example field-in-field) were not used. Intensity-modulated radiotherapy (IMRT) was not used because our institution has no established policy of applying this treatment modality in patients with lung cancer. IMRT is not available in all institutions and is used for lung cancer only at a limited number of institutions. As 3D-CRT is an alternative treatment option for patients who are not suitable candidates for SBRT, it is reasonable to perform 3D-CRT at hospitals where SBRT, including IMRT, is not available. Thus, we believe that presenting data based on conventional 3D-CRT is both important and clinically relevant. The superposition method for the algorithm was used to calculate the irradiation dose. The minimum and maximum doses according to the PTV were 95 and 107 %, respectively (in the case of a large PTV, delivering a minimum dose of 90 % was acceptable). Elective nodal irradiation was not adopted for all patients. More recently, as long as no respiratory disturbance is present, irradiation is administered in our center in a single fraction at a total dose of more than 5 Gy, irrespective of tumor site and size.

### Evaluation of the initial clinical response and toxicity

All patients underwent routine X-ray imaging and tests for serological tumor markers at 1 month after radiotherapy and every 3 months thereafter. In the event of poor X-ray results and positive tumor markers, a lung CT was performed. Routine lung CT was performed every 3–6 months following the completion of radiation therapy. A complete response (CR) was defined as the disappearance of all measurable disease and the absence of newly-developing lesions for 4 weeks. For measurable disease, a partial response (PR) was defined as a reduction in more than 30 % of the sum of the cross-sectional diameters of all measurable lesions over 4 weeks. Progressive disease (PD) was defined as either an increase of greater than 20 % of the sum of the cross-sectional diameters of all assessable lesions in 4 weeks or the appearance of new lesions. Stable disease (SD) was determined when there was an insufficient increase in tumor size to qualify as PD. SD also included the situation in which a tumor decreased in size enough to no longer meet the definition of PD. Responses were scored when the treatment was most effective. Local recurrence was defined as changes similar to those of PD. When tumor growth was difficult to assess because of radiation pneumonitis, tumor markers measured each month were used as reference values, and the date of recurrence was determined as the first day when levels began to rise. Adverse events were defined according to the Common Terminology Criteria (CTC) for Adverse Events, version 4.0, with toxicity graded as mild (CTC Grade 1), moderate (CTC Grade 2), severe (CTC Grade 3), or life-threatening (CTC Grade 4) [23].

### Statistical analysis

Overall survival was calculated as the interval from the first day of treatment to the date of death or the last follow-up before June 2014. If they reach the end of the following period without having an event, patients were censored. Local tumor control (LC) was calculated as the period from the first day of treatment until local relapse. Patients who died with no evidence of recurrence were censored. Survival curves were generated using Kaplan–Meier analysis. Univariate survival comparisons were performed using the log-rank test. The analyzed prognostic factors for survival were age (<75 vs. ≥75), PS (≤1 vs. ≥2), T-stage (T2 vs. T3), location (lower lobe vs. middle and upper lobes, near the pulmonary hilum vs. peripheral), and pathology (squamous vs. adeno). Independent variables that appeared statistically significant on univariate analysis were tested by multivariate analysis. *P* < 0.05 indicated statistical significance. All calculations and survival displays were conducted using SPSS 15.0 J statistical software (SSPS Inc., Chicago, IL, USA).

### Patient consent

The present study was a retrospective analysis of patient diagnostic and treatment data. Written informed consent was obtained from all patients, who were informed that their data would be included in the study.

## Results

### Patient and treatment characteristics

Baseline patient characteristics are listed in Table 1. Stage T2a NSCLC was present in 18 patients, whereas nine patients were diagnosed with stage T3 disease (thoracic invasion in eight patients and additional tumor nodules in the same pulmonary lobe in one patient). SBRT was proactively performed in most patients with stage T1 disease, and these patients were not included in the present study. The median tumor diameter was 31 mm (range, 25–60 mm) in T2 cancer and 42 mm (range, 26–60 mm) in T3 cancer. Tumors near the hilum, i.e., those defined as being located within 2 cm from the pulmonary hilum, were detected in 8 patients. Tumors growing in the lower lobes were found in 11 patients. Dose fractionation was higher in the most recent cases, with the highest dosage of 6 Gy per fraction administered to two patients. The most common dose was 5 Gy per fraction, administered to 15 patients. The treatment was given in three fractions per week in one patient because of the patient’s general condition, and all other patients received conventional radiotherapy administered in a single fraction per day. No patients underwent accelerated HFRT. To compare the effects of different protocols with different fraction sizes and total doses, the BED was adopted using a linear quadratic model. The α/β ratio was assumed to be 10 for acute effects on normal tissue and lung tumors. The BED ranged from 67.2 to 96.0 Gy. The median BED was approximately 80 Gy. When 80 Gy was used as a cut-off value, BED was less than 80 Gy in 14 patients and more than 80 Gy in 15 patients.

**Table 1:** Clinical characteristics of patients

Characteristics		Number
Patients		29
Age, median (range)		76 (56–89)
<75		
≥75		
Gender		
	male	20
	female	9
PS		
	0	2
	1	22
	2	5
T-stage		
	T2a	18
	T2b	2
	T3	9
Location of the tumor 1		
	lower lobe	11
	middle, upper lobe	18
Location of the tumor 2		
	near the pulmonary hilum	8
	Peripheral	21
Pathology		
	squamous carcinoma	17
	adenocarcinoma	12
Dose (Gy), median (range)		60 (48–60)
	48Gy, 3Gy/f	1
	50Gy, 5Gy/f	3
	54Gy, 6Gy/f or 3Gy	2
	60Gy, 3Gy/f	2
	60Gy, 4Gy/f	8
	60Gy, 5Gy/f	10
	60Gy, 6Gy/f	3
BED (Gy), median (range)		84 (67.2-90)
	<80 Gy	14
	≥80 Gy	15
Fraction size (Gy)		
	3	7
	4	5
	5	15
	6	2
Chemotherapy		
TS-1	neoadjuvant	1
	concurrent	1
	adjuvant	1
UFT	concurrent	1

*Abbreviations*: *BED* biological effective dose

### Patient survival, response, and tumor recurrence

**Fig. 1:**
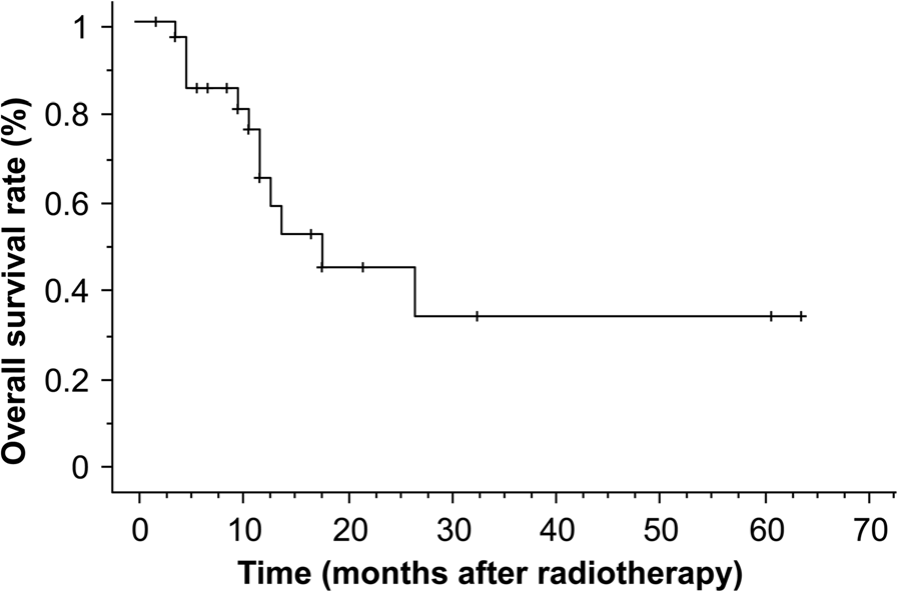
The median survival time following radiotherapy with three-dimensional conformal radiation therapy was 17 months (range, 1–64 months), and the estimated 3-year survival rate was 38 %

**Fig. 2:**
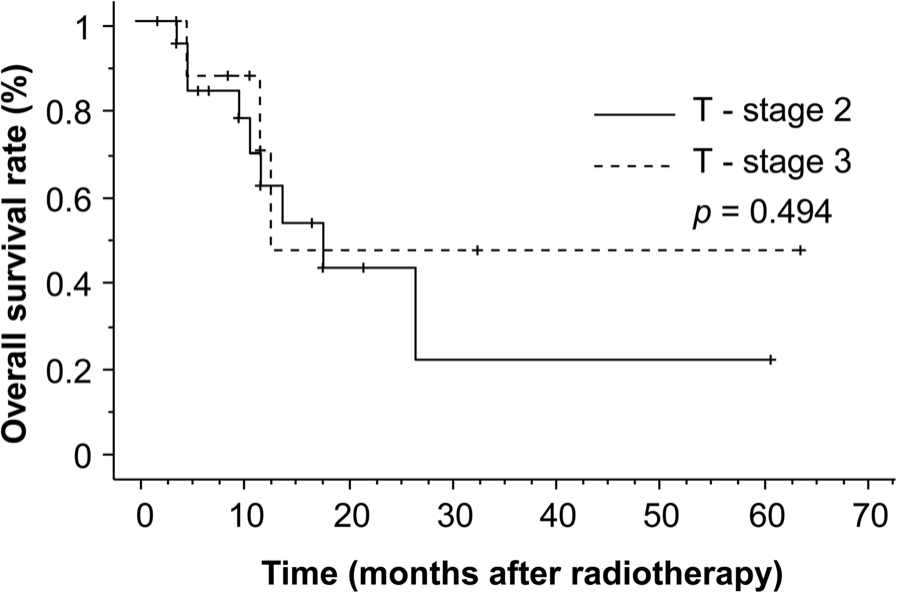
Comparison of overall survival (OS) from radiotherapy with three-dimensional conformal radiation therapy. There was no significant difference in OS between patients with T2 and T3 non-small cell lung cancer

**Fig. 3:**
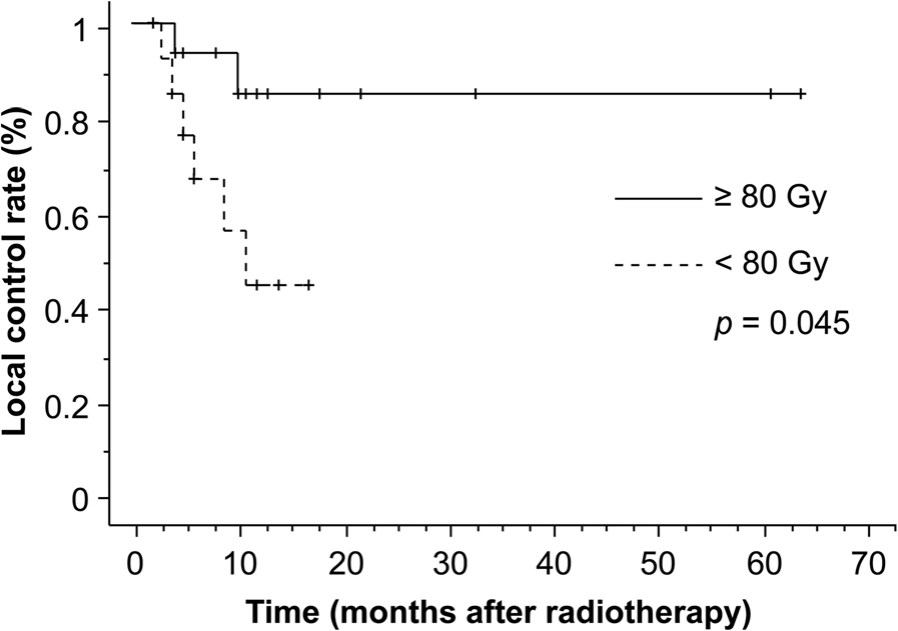
Comparison of local control from radiotherapy with three-dimensional conformal radiation therapy. There was a significant difference between patients who received a dose of <80 Gy and those treated with a dose of ≥80 Gy (*P* = 0.045)

**Fig. 4:**
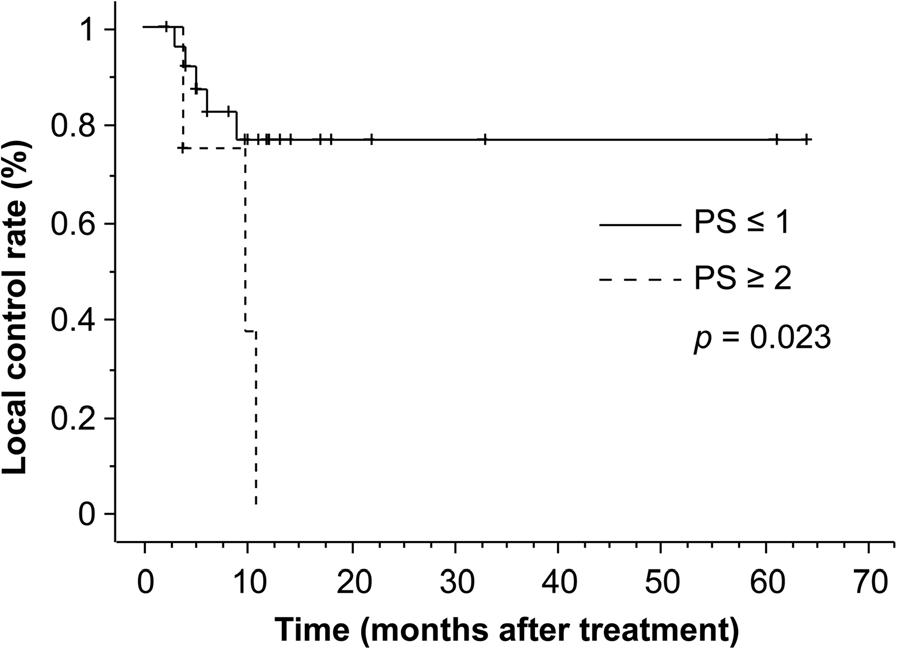
Comparison of local tumor control (LC) from radiotherapy with three-dimensional conformal radiation therapy. There was a significant difference in LC between patients with a performance status (PS) of ≤1 and those with a PS of ≥2 (*P* = 0.023)

The median duration of observation was 17 months (range, 1–64 months). CRs and PRs were recorded in four (13.8 %) and 13 patients (44.8 %), respectively. SD occurred in four patients (13.8 %), and PD occurred in eight patients (27.6 %). The overall response rate was 58.6 %. The 1- and 3-year overall survival rates were 65.8 and 33.8 %, respectively (Fig. 1). There was no significant difference in OS between patients with T2 and T3 (Fig. 2). The 1- and 3-year survival rates and prognostic factors identified by univariate analysis are listed in Table 2. However, there were no significant differences observed for any factors. During the follow-up, 11 patients died. The causes of death were primary disease in seven patients, brain metastasis in two, other diseases in two, and unknown in one. The 1- and 3-year cause-specific survival rate was 77.3 % and 49.7 %, respectively. Local tumor recurrence was observed in eight patients (two patients’ imaging evaluation was difficult due to radiation pneumonitis, and local recurrence was identified by elevated tumor markers without metastasis to other sites) and the 1-year LC rate was 66.1 %. Upon univariate analysis, the BED (≥80 Gy vs. <80 Gy) and performance status (≤1 vs. ≥2) were significantly related to LC. Figures 3 and 4 illustrate the LC curves for NSCLC for each patient group. Upon multivariate analysis, only BED was a significant factor for LC (*P* = 0.037; hazard ratio = 10.10; 95 % confidence interval = 1.150–88.67) (Table 3).

**Table 2:** Univariate analysis to identify factors that affect survival and 1- and 3-year overall survival rates

Variables	*p* value	1-year	3-year
Age			
<75	0.958	58.3 %	38.9 %
≥75		70.3 %	35.2 %
PS			
≤1	0.163	70.5 %	45.7 %
≥2		33.3 %	−
T-stage			
T2	0.494	62.4 %	21.4 %
T3		72.9 %	48.6 %
Location 1			
Lower lobe	0.250	39.4 %	19.7 %
Middle and upper lobe		81.9 %	19.0 %
Location 2			
Near the pulmonary hilum	0.235	75.0 %	75.0 %
Peripheral		61.2 %	17.5 %
Pathology			
Squamous	0.728	61.4 %	28.1 %
Adeno		79.5 %	53.0 %
BED (Gy)			
<80 Gy	0.087	55.9 %	−
≥80 Gy		76.2 %	63.5 %

*Abbreviations*: *BED* biological effective dose

**Table 3:** Univariate and multivariate analysis of factors that affect local control (LC)

Variables	*p* value	Multivariate analysis		
		HR	95%CI	*p*
Age (years)	0.333	0.893	0.125–6.393	0.910
<75 vs. ≥75				
PS	0.023	9.773	0.778–122.7	0.077
≤1 vs. ≥2				
T-stage	0.700	0.212	0.017–2.671	0.230
T2 vs. T3				
Location 1	0.966	6.992	0.956–51.16	0.055
Lower lobe vs. middle and upper lobe				
Location 2	0.330	2.102	0.140–31.55	0.591
Near the pulmonary hilum vs. peripheral				
Pathology	0.505	0.303	0.034–2.680	0.283
Squamous vs. adeno				
BED (Gy)	0.045	10.10	1.150–88.67	0.037
<80 Gy vs. ≥80 Gy				

*Abbreviations*: *BED* biologically effective dose

### Radiation toxicity

Among acute toxicities, grade 1 pneumonitis was observed in three patients who received irradiation at a BED of 80 Gy or more and in two patients with a BED of less than 80 Gy. No patient was given oral steroid treatment or oxygen therapy. No patient developed esophagitis or macrovascular disease. No severe adverse events were observed.

## Discussion

This retrospective study was performed to investigate a possible a role for 3D-CRT as a monotherapy for patients who refuse or could not tolerate surgical therapy with early-stage NSCLC. In this initial study, the 1-year survival rate for patients was 65.8 %, and the 3-year survival rate was 33.8 %. A significant difference in LC was observed with a BED of 80 Gy or more, and the side effects were deemed acceptable. Currently, surgery is the main treatment method for localized NSCLC, and it has achieved favorable outcomes. The Japanese Joint Committee of Lung Cancer Registry investigated prognosis in 6644 patients who underwent resection for NSCLC by histologic type [24]. The 5-year survival rates for patients with clinical stages 1A and 1B NSCLC were 72 and 50 %, respectively. The 3-year survival rates for those with clinical Stages 1A and 1B were 82 and 63 %, respectively [24]. However, a limited number of patients have operable early-stage tumors, and elderly patients are at high risk from complications of surgery. The high long-term costs of surgery and postoperative care justify radiotherapy as a minimally invasive alternative treatment in NSCLC [4]. In our study, the 3D-CRT outcomes for survival was below what would be expected for potentially curative surgery, but none the less, represent an alternative to patients that lack a surgical option.

Conventional methods of administering radiation at a dose of 1.8 or 2 Gy in a single fraction for a total of 60 to 66 Gy results in an LC rate of 50 % and a 3-year survival rate of 20 to 30 %, representing unsatisfactory outcomes [6–9]. Increasing the dosage prolongs the treatment period, which in turn results in increased patient burden and medical costs. Recent improvements in radiation therapy techniques have led to the widespread popularity of SBRT as an alternative therapy to radical surgery in NSCLC. In a recent systematic review, the 5-year survival rate in patients undergoing SBRT was estimated at 47 % (range, 18–78 %) and the LC rate was 80–100 % [25]. In patients with early-stage NSCLC, it is currently possible to achieve results comparable to those of surgery [13]. Thus, SBRT should be considered for patients with localized NSCLC who are inoperable. SBRT requires greater precision and accuracy than conventional HFRT and 3D-CRT, and it must be performed using a strict protocol that may not be available in all institutions [15]. A recent survey conducted in Japan reported that 44 % of replying institutions did not utilize SBRT [26]. SBRT can be deemed difficult due to varying patient factors, such as when stable respiration is not possible, when the tumor is close to major blood vessels or the hilar region, when respiratory function is poor, and when the patient is in poor general condition. In such instances, 3D-CRT could be an alternative treatment option. Despite the potential role for 3D-CRT in the treatment of NSCLC, there have been few treatment outcome studies. Past reports indicate that the radiation field varies from a field encompassing the tumor plus margin to a field in which elective nodal irradiation is performed, and irradiation techniques and schedules differ between reports [27]. Patients with T2 or T3 NSCLC have a higher incidence of lymph node metastasis, and therefore, many receive concurrent anticancer agents. Thus, irradiation monotherapy is limited to patients with T2 or T3 cancer without lymph node metastasis, and the irradiation field involves the tumor site only. At our institution, a total of 525 patients received radiotherapy for primary lung cancer during the observation period of January 2005 to June 2014. Of these patients, 29 with T2 or T3 localized lung cancer (approximately 5.5 %) underwent 3D-CRT monotherapy with the irradiation field set as the tumor plus tumor margin only. We believe that the results of the present study provide valuable data for future 3D-CRT treatment strategies. In the present study, the 3-year survival period following 3D-CRT for localized lung cancer was comparable with, or better than, those of previous reports [6, 8, 28]. This may be attributed to the difference in radiation distribution. Previous reports described irradiation performed with three to five portals, whereas more than five irradiation portals are used at our institution [6, 8, 28]. The relatively small tumor size in the present study may also have contributed to the findings, with a T staging of T2a in 18 patients and T2b in two patients. Another reason for the findings in this study could be that patients who underwent thorough staging by PET were included. The reason that no difference in survival was observed between T2 and T3 patients is believed to be comparable median tumor diameter in the two groups of patients (T3, 42 mm and T2, 31 mm). It has been reported that patients with T2a (stage IB) disease who underwent SBRT had a 3-year survival rate of 63 % [29]. Based on our results, 3D-CRT appeared to have inferior outcomes to SBRT in patients with early-stage NSCLC. Thus, one conclusion of this present study is that although 3D-CRT may be a second treatment option for patients with early-stage NSCLC who are unable to undergo SBRT, it may not be an alternative treatment. A Japanese multi-institutional, retrospective survey revealed that a BED greater than 100 Gy resulted in significantly improved patient survival and LC than a BED of less than 100 Gy when SBRT was used to control stage I NSCLC [12]. Concerning BED, a radiation dose exceeding 100 Gy resulted in different control rates in T1 and T2 disease, whereas in the event of T2 lung tumors, a BED greater than 120 Gy was required [12]. In this study, LC was significantly improved in patients administered a BED of 80 Gy (range, 67.2–96.0 Gy). Previous reports of the use of 3D-CRT described treatment with a 3–3.5-Gy single fraction [16, 19]. More recently, it has been reported that good LC has been achieved with irradiation using a single 4-Gy fraction, and adverse events were within permissible ranges [20]. The total dosage is also reported to be an important factor [30]. This study has demonstrated that a BED greater than 80 Gy is an independent and significant prognostic factor in LC, using a linear-quadratic model and an α/β ratio of 10 to determine the acute effect on tumor and normal tissues. In a previous study using conventional fractionation, improved local PFS was observed in a subgroup of patients with no nodal disease who were receiving >73 Gy during 3D-CRT [30]. The LC rate in the present study is believed to be comparable with these earlier results. It has previously been reported that radiotherapy toxicities of CTC Grades 3–5 develop in 20 % of patients receiving SBRT with a single fraction that exceeds 20 Gy [31]. This same study concluded that the regimen should not be used for patients with tumors near the central airways because of excessive radiation toxicity [31]. Even for SBRT using approximately 12 Gy delivered in a single fraction, a tumor located near a major vessel or serial organ poses a concern for the occurrence of adverse events. In the present study, no severe adverse events (CTC Grade 2 or more) were observed with 3D-CRT and with irradiation given at a BED of more than 80 Gy. A previous report supports these findings, showing that no severe adverse events developed in 3D-CRT with a BED over 90 Gy [19]. The results of this study support the view that to improve LC in early-stage NSCLC, if the patient’s respiratory function and general physical condition allow, the dose per fraction should be increased, and if possible, the BED should exceed 80 Gy. Furthermore, because this study has confirmed that there are no severe radiation toxicities associated with this regimen, it is possible that the dosage may be increased further. This consideration and other aspects of the role of 3D-CRT as monotherapy in early-stage NSCLC require investigation in further controlled studies with larger patient numbers. Because of current advances in therapy for NSCLC, as well as the increasing numbers of patients treated with combined anticancer agents, the number of patients receiving 3D-CRT within one center will be limited.

Our study limitations include lack of long-term follow up and small number of patients, because of which the results of multivariate analysis were unclear. This limitation may be addressed in the future by studies involving multiple centers in multiple countries.

## Conclusions

The retrospective nature of this study and small number of patients who were available within a single center do not detract from the value of these preliminary findings. This study has demonstrated that 3D-CRT may be used as a monotherapy for patients with T2 or T3 NSCLC as a second treatment option for patients unable to receive SBRT. Although the therapeutic efficacy outcomes in this small study were inferior to those reported for SBRT, the safety outcomes were comparable. Furthermore, to improve LC in early-stage NSCLC, this study found that the BED should exceed 80 Gy, and it is possible that this dosage could be increased even further. These findings support a role for 3D-CRT as a treatment option for patients who refuse or could not tolerate surgical therapy with early-stage NSCLC, and they may form the basis for future, larger controlled studies on 3D-CRT as a monotherapy option for early-stage NSCLC.

## Abbreviations

BED: Biologically effective dose
CTC: Common Terminology Criteria
CR: Complete response
CT: Computed tomography
CTV: Clinical target volume
HFRT: Hypofractionated radiotherapy
ITV: Internal target volume
MRI: Magnetic resonance imaging
MST: Median survival time
NSCLC: Non-small cell lung cancer
PET: Positron emission tomography
PR: Partial response
PTV: Planning target volume
SD: Stable disease
SBRT: Stereotactic body radiotherapy
3D-CRT: Three-dimensional conformal radiation therapy
